# Patterns of sequencing coverage bias revealed by ultra-deep sequencing of vertebrate mitochondria

**DOI:** 10.1186/1471-2164-15-467

**Published:** 2014-06-12

**Authors:** Robert Ekblom, Linnéa Smeds, Hans Ellegren

**Affiliations:** Department of Ecology and Genetics, Uppsala University, Uppsala, SE-75236 Sweden

**Keywords:** Next generation sequencing, Sequencing bias, Error rate, SSE, mtDNA

## Abstract

**Background:**

Genome and transcriptome sequencing applications that rely on variation in sequence depth can be negatively affected if there are systematic biases in coverage. We have investigated patterns of local variation in sequencing coverage by utilising ultra-deep sequencing (>100,000X) of mtDNA obtained during sequencing of two vertebrate genomes, wolverine (*Gulo gulo*) and collared flycatcher (*Ficedula albicollis*). With such extreme depth, stochastic variation in coverage should be negligible, which allows us to provide a very detailed, fine-scale picture of sequence dependent coverage variation and sequencing error rates.

**Results:**

Sequencing coverage showed up to six-fold variation across the complete mtDNA and this variation was highly repeatable in sequencing of multiple individuals of the same species. Moreover, coverage in orthologous regions was correlated between the two species and was negatively correlated with GC content. We also found a negative correlation between the site-specific sequencing error rate and coverage, with certain sequence motifs “CCNGCC” being particularly prone to high rates of error and low coverage.

**Conclusions:**

Our results demonstrate that inherent sequence characteristics govern variation in coverage and suggest that some of this variation, like GC content, should be controlled for in, for example, RNA-Seq and detection of copy number variation.

**Electronic supplementary material:**

The online version of this article (doi: 10.1186/1471-2164-15-467) contains supplementary material, which is available to authorized users.

## Background

Many applications of high throughput sequencing, such as expression profiling [1], splicing inference [2], copy number variation (CNV) identification [3] and repeat element annotation [4] rely on observed variation in the depth of sequencing coverage within the genome or transcriptome. In addition, variance in coverage is of importance to *de-novo* assembly pipelines and read mapping strategies, since local regions of unusually high coverage may be interpreted as duplications or masked as repeat elements [5, 6]. As a consequence, applications such as these may be impaired by random fluctuation and systematic bias in sequencing coverage across the genome. However, the details and extent of these effects are currently not well understood.

It is known that the PCR step involved in sequencing-by-synthesis methods introduces coverage bias related to GC content [7–9], possibly due to the formation of secondary structures of single stranded DNA [10]. Such GC dependent bias is seen on a wide variety of scales ranging from individual nucleotides to complete sequencing reads and even large (up to 100 kb) genomic regions [11]. Systematic bias could also be introduced during the DNA fragmentation step or caused by DNA isolation efficacy [12], local DNA structure, variation in sequence quality and map-ability of sequence reads [13]. Some efforts have been made to control for these biases during downstream computational analyses in various NGS (next generation sequencing) applications [13–16], and laboratory protocols have also been developed to reduce this problem [17].

In addition to variation in coverage, there may be sequence dependent variation in nucleotide specific error rates. Such systematic patterns of sequencing errors can also have consequences for downstream applications as errors may be taken for low frequency SNPs, even when sequencing coverage is high [18]. GC rich regions and sites close to the ends of sequence reads typically show elevated errors rates [19] and it has also been shown that certain sequence patterns, especially inverted repeats and “GGC” motifs are associated with an elevated rate of Illumina sequencing errors [10]. Such sequence specific miscalls probably arise due to specific inhibition of polymerase binding [19]. Homopolymer runs cause problems for technologies utilising a terminator free chemistry (such as Roche 454 and Ion Torrent), and specific error profiles exist for other sequencing technologies as well [20]. The effects of such technology specific error patterns on sequencing coverage and read assembly algorithms remains poorly described. As sequencing reads with high error rates are more likely to be removed during trimming stages, regions with high error rates may also get decreased sequencing coverage [21].

Mitochondrial DNA (mtDNA) is abundant in most cell types and whole genome, exome or transcriptome data from high throughput sequencing can therefore be efficiently mined for complete mitochondrial sequences, as shown in a number of recent studies [22–29]. Due to the haploid nature of the mitochondrial genome, such data is also comparatively easy to assemble and analyse. Here we take advantage of ultra-deep sequencing of mtDNA from two vertebrate species to evaluate fine scale variation in coverage and patterns of sequencing errors. We utilise data from several independently sequenced individuals in order to describe systematic coverage bias across the mtDNA sequence. Due to the extreme depth of our sequencing data (>100,000X), it is possible to provide very precise estimates of coverage bias and sequencing error rates. Furthermore, a comparison in sequence coverage between two distantly related species (one bird and one mammal) allows us to evaluate to what extent sequencing bias is conserved across evolutionary distant lineages.

## Results and discussion

### Sequencing the wolverine mitochondrial DNA

About 20 million genome sequence reads from a single individual mapped against the wolverine mtDNA sequence (Table 1). The entire 16,537 bp consensus mtDNA sequence was covered by an average sequencing depth of 111,757X (Additional file 1: Figure S1). Thus, 2% of all quality trimmed genome sequence reads mapped to mtDNA, even though the mitochondrial genome only represents ~0.0005% of the total genome. Mitochondria may be expected to be especially common in tissues with high oxygen demands [30, 31], and the use of muscle tissue for genome sequencing could thus potentially explain the high coverage of mtDNA sequences in our data.

**Table 1:** **Information about sequencing coverage and read mapping for the sampled individuals**

	Total amount of trimmed sequencing data: no reads (no base pairs)	Number of reads mapping to mtDNA	Average mtDNA sequencing coverage
*Wolverine:*			
Main individual	975 million reads (93.0 Gbp)	19,545,210	111,757X
Additional ten individuals (mean)	152 million reads (16.4 Gbp)	619,489	4,168X
*Flycatcher:*			
Main individual	952 million reads (93.8 Gbp)	23,803,990	119,847X

Our consensus mtDNA sequence from the wolverine differed from the previously published wolverine mtDNA sequence [32] by 23 SNPs and 12 indels. Given the high sequencing coverage underlying the consensus sequence, we are fairly confident that it is error free. Moreover, no mitochondrial sequence variation in non-repetitive regions was detected in sequencing of 10 additional samples from different parts of the same Scandinavian population (see below). Since the previously sequenced individual was allegedly also sampled from the Scandinavian population [32], the observed discrepancies are most likely explained by sequencing errors in the NCBI entry. Complete mtDNA homogeneity has previously been found when analysing a small region of the mitochondrial genome in Scandinavian wolverines [33].

A k-mer count (k = 25) of mtDNA reads showed a peak at a multiplicity at 52,000 (the mean k-mer coverage of the sequencing data), representing a mean depth of sequencing coverage of 68,000X (see Materials and Methods for details of this calculation). Based on the shape of the bimodal distribution (Additional file 1: Figure S2), k-mers with multiplicity less than 8,000 were inferred to be the result of sequencing errors. Out of a total of 861 million k-mers in the data, 24 million were thus considered to be the result of sequencing errors. This gives an average per-base error rate of 0.11% across the whole mtDNA data.

### Variation in coverage across the mtDNA sequence

**Figure 1:**
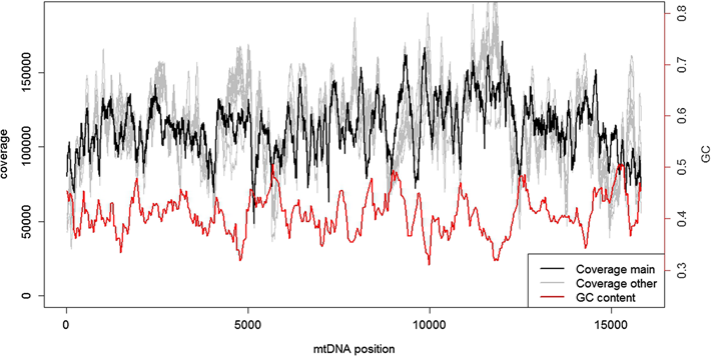
**mtDNA Sequencing coverage.** Sequencing coverage of the wolverine mtDNA for the individual sequenced at high depth (in black) and 10 additionally sequenced individuals (in grey, scaled to the same mean coverage as the main individual). Sliding window estimate of GC content in red (window size: 250 bp, step length: 37 bp).

The per-site depth of sequencing coverage across the wolverine mtDNA ranged between 48,660X and 171,260X (Figure 1). Given the extreme depth of covarage, stochastic variation in measurement should be negligible and the more than three-fold difference in coverage among sites observed should therefore be related to inherent characteristics of the sequence. We calculated sequencing coverage based on the number of individual sequencing reads covering each nucleotide. Alternatively, coverage can also be calculated based on the whole DNA fragments (thus also including the non-sequenced parts between two paired reads) [11]. We found that the pattern of coverage variation was strikingly similar for read and fragment coverage in our data (Additional file 1: Figure S3), the only qualitative difference being a slightly higher overall coverage for the fragment counts compared to the read counts.

**Figure 2:**
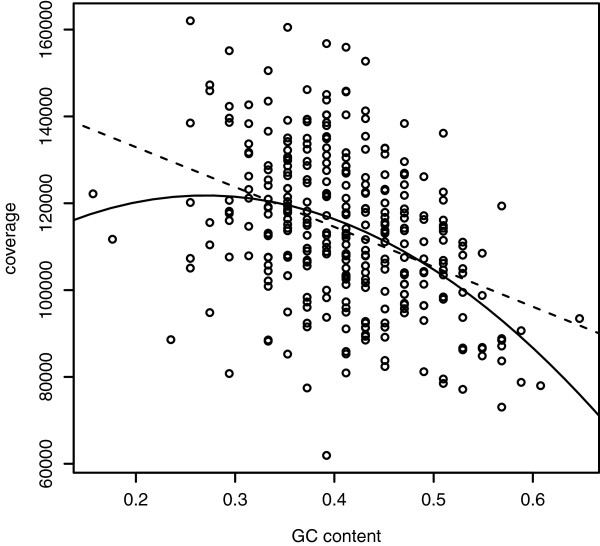
**GC dependent sequencing coverage.** The relationship between GC content and sequencing coverage in wolverine (from the deep sequenced individual, estimates based on non-overlapping 50 bp windows). Linear (dashed) and quadratic (solid) regression lines are included.

There was a clear relationship between local (50 bp windows) GC content and sequencing coverage (linear regression, F = 58.07, R^2^ = 0.16, df = 313, p < < 0.001; quadratic regression, F = 34.47, R^2^ = 0.18, df = 312, p < < 0.001; Figure 2). As in previous studies [11, 17], we found some evidence that this relationship is non-linear, indicated by the quadratic regression having a slightly better fit to the data than the linear regression (ΔAIC = −7.3, Figure 2). However, the peak of the quadratic regression lies very close to the lower extreme of our data range (at GC = 27.0%), meaning that the relationship between GC content and coverage is essentially negative for most of the sequence. This finding is strikingly different compared with nuclear genome sequences obtained using Illumina technology, where the peak of sequencing coverage typically occur at a GC content close to 50% e.g. [34, 35]. In our data, inter-genic regions had lower sequencing coverage than genes, and rRNA genes had lower coverage than protein-coding genes and tRNA genes (Additional file 1: Figure S4, Additional file 1: Table S1). This is in line with previous studies showing increased coverage on exons [13].

Individual G and C nucleotides had lower average sequencing coverage (mean = 111,750) compared to A and T nucleotides (mean = 114,853; t = 10.51, df = 13,765, p < < 0.001, Additional file 1: Figure S4), also when controlling for GC content of the surrounding region (Additional file 1: Table S1). Under-representation of GC-rich regions has been suggested to be a result of bias in the initial PCR steps during library preparation [20, 36] and of miscalls originating from the sequencer [10]. These local fluctuations in sequencing coverage may affect applications where coverage is used as a proxy for biological phenomena. For instance regions of high coverage (due to low GC content) could be more likely to be interpreted as genomic duplications in CNV analyses [37]. Moreover estimates of expression levels of GC-poor genes may be inflated in RNA-Seq experiments [38]. As GC-rich regions are common around promoters and transcription start sites, under-representation of sequencing there may hamper biological interpretation and annotation of low coverage genome data [20].

### Reproducibility of sequencing coverage

Strong repeatability in the pattern of heterogeneity in coverage across a sequence would evidence that the variation is mainly due to inherent characteristics of particular sites or regions of that sequence, rather than representing stochastic variation in library preparation and/or amplification. One means to further test this is to investigate among-individual consistency in sequencing coverage. We therefore sequenced 10 additional wolverines to a mean trimmed read depth of 5.5X coverage for nuclear DNA and 4,200X coverage for mtDNA. The per-site variation in coverage of mtDNA was again considerable, ranging between three-fold and six-fold for the 10 individuals. Variation was highly correlated between the individual sequenced at high coverage and the additional 10 individuals (Spearman’s r ranging from 0.53 to 0.68, df = 15,786, p < < 0.001, Figure 1). The correlation was stronger when comparing among the 10 additional individuals (r_S_ ranging from 0.70 to 0.91; paired t-test, t = −14.67, df = 9, p < 0.001). Several peaks and dips of coverage were concordant among the independently sequenced individuals (for example at around positions 2,000, 7,000 and 9,000; Additional file 1: Figure S5), even on a very fine scale. Other regions (around positions 4,600, 11,400 and 15,500) showed more variation among individuals (Additional file 1: Figure S6). It is thus evident that although there is some random fluctuations in coverage, a large proportion of the variation is due to systematic and sequence dependent biases.

### Conservation of sequencing coverage bias across two distant vertebrate taxa

**Figure 3:**
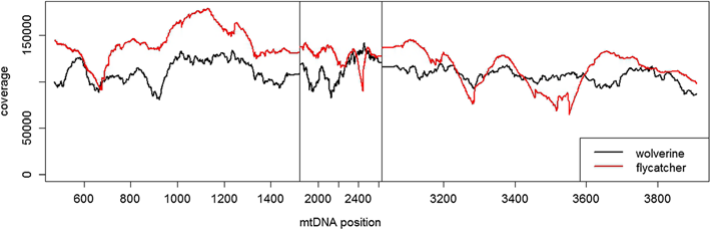
**Conservation of sequencing coverage patterns across taxa.** Comparison of sequencing coverage of wolverine and flycatcher in three homologous regions of the mtDNA.

In order to further investigate the repeatability in variation in coverage we benefitted from the strong conservation in the genomic organisation of animal mtDNA. Using avian genome sequencing data from a single collared flycatcher with a nuclear genome coverage of 85X, we analysed variation in coverage of mtDNA in a similar way as above. There was an even higher variation in sequencing depth, with per-site coverage ranging between 29,010X and 200,900X. As for wolverine, a clear relationship between GC content and coverage was found for flycatcher (quadratic regression, F = 18.52, R^2^ = 0.097, df = 326, p < 0.001). We then used regions of the mitochondrial genome that were highly conserved (73% sequence similarity) between wolverine and flycatcher (three regions of continuous sequence with a total of 2,594 bp, containing both rRNA genes and the protein coding gene NAD1) and found a positive correlation between coverage in wolverine and flycatcher at the nucleotide level (r_S_ = 0.28, df = 2,592, p < < 0.001, Figure 3, Additional file 1: Figure S7). The pattern of sequencing coverage bias was thus strikingly conserved even across two distantly related vertebrate lineages.

### Site-specific variation in sequencing errors

For all positions (and in all sequenced individuals) in the wolverine mtDNA sequence we found that a small proportion of the reads had an aberrant nucleotide compared to the reference sequence. A large majority of sites have a non-reference nucleotide in ≈ 0.1% of the reads with a similar distribution across all eleven sequenced individuals. We interpret these alternative nucleotides as sequencing errors (rather than true mutations) based on their relatively low frequency and the fact that they were dispersed across all sequenced individuals and across the whole mtDNA sequence. An observed transition/transversion ratio close to one (Additional file 1: Table S2), distinctly different from what is normally observed in diversity or divergence data, further supports this interpretation. The estimated mean error rate (0.11%, see below) is very close to our expectation based on the k-mer count analysis (see above) and it is also in line with the specifications for HiSeq sequencing (http://www.illumina.com), representing a Qphred score of 30; and to that found in other studies after quality filtering [21]. Six nucleotides had a very high number of one specific non-reference allele (1.3-67.9%) in a single individual only (Additional file 1: Table S3, Additional file 1: Figure S8), with all other individuals having a low (less than 0.1%) number of non-reference nucleotides at this site. We interpret this as evidence for heteroplasmy, i.e. mutations in the pool of mtDNA molecules transmitted between generations [39].

The mean site-specific error rate (measured as the count of a non-reference nucleotide divided by total sequencing depth at that position) of the wolverine individual sequenced at high depth was 0.11%, with a range between 0.028% and 2.45%. Sequencing errors thus occurred at all positions of the mitochondrial genome. G and C nucleotides had a slightly higher mean error rate compared with A and T nucleotides (t = −10.14, df = 15,550, p < < 0.001, Additional file 1: Table S2). Sequence specific errors (SSE) were previously found to be common in Illumina HiSeq reads with the highest rates seen at the motif “GGC” [10] and, in particular, “GGCNG” [36, 40]. Our data support these findings. All three sites with error rates above 1% included the sequence motif “CCNGCC” (a one-base pair extension of the reverse complement of the above) directly downstream of the high error position (Additional file 1: Figure S9). SSE are likely to bias estimates of sequence diversity as they will lead to falsely inferred SNPs in specific error prone sites. An efficient way of overcoming this problem would be to consider on which strand SNPs are detected (only calling SNPs if the variation is seen on both strands), as only reads from one direction should be affected by the increased error rate [19]. It is however, important to note that this strategy requires sufficient sequencing coverage of both strands at the variable nucleotide position.

**Figure 4:**
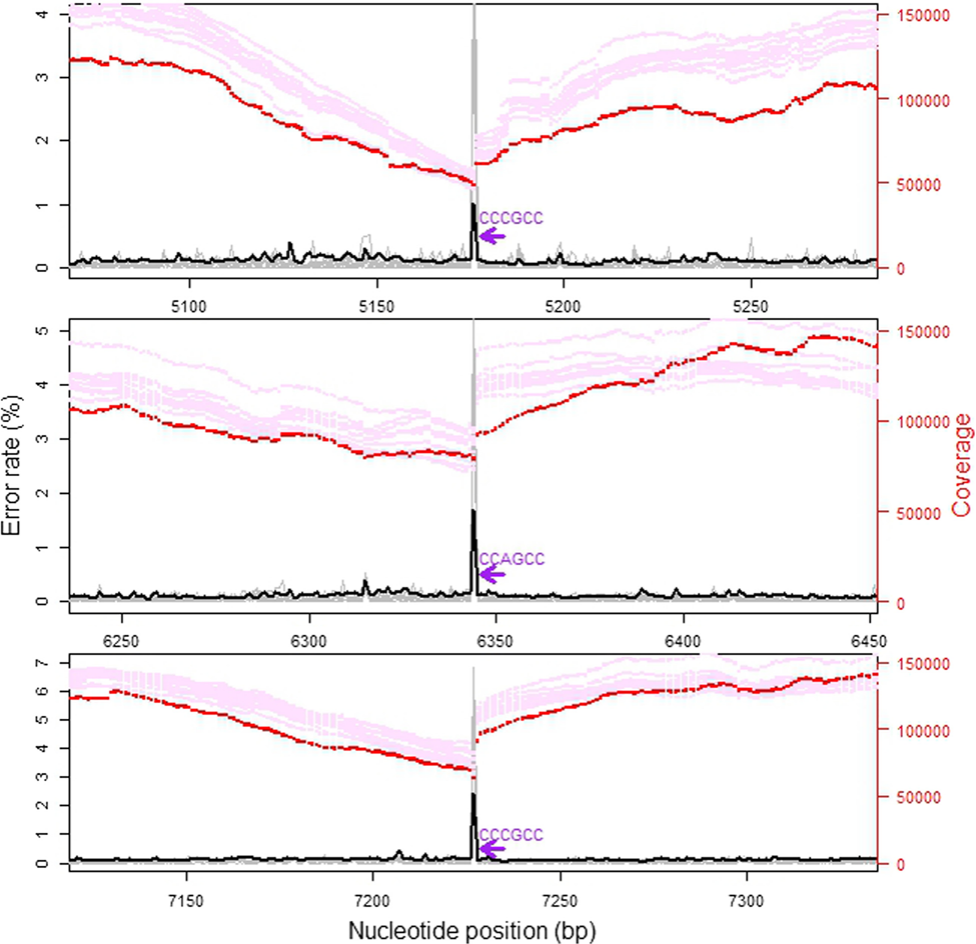
**Sequencing coverage around peaks of sequencing errors.** Sequencing coverage (main individual in red, additional individuals, scaled to the same mean as the main individual, in pink) in the regions with peaks of sequencing error rates (main individual in black, additional individual in grey) associated with the sequence motif “CCNGCC” (purple arrow). Note the coinciding location of marked drops of sequencing coverage exactly at the sequencing error peak.

There was a negative correlation between sequencing coverage and site specific sequencing error rate (r_S_ = −0.083, df = 15,786, p < < 0.001). When investigating areas directly downstream of “GGCNG” (and the reverse complement of that motif), coverage clearly decreased with proximity (within 100 bp) to the error prone motif (r_S_ = 0.21, df = 6,570, p < < 0.001). Inspecting the details of the regions surrounding the three nucleotide positions with the highest error rates (>1%), it is obvious that there is a marked drop in sequencing coverage exactly at, and upstream of, the high-error base (Figure 4). Miscall frequency may thus also be directly linked to the observed bias in sequence coverage. This link could potentially arise as a consequence of read trimming. If reads (or parts of reads) from SSE regions have lower quality scores, they will be more likely to be trimmed away during initial read filtering steps. To explore this, we also investigated coverage based on raw (untrimmed) reads (Additional file 1: Figure S3). The coverage variation pattern seen after mapping the raw reads was strikingly similar to the trimmed coverage data (but with higher overall coverage levels and with even stronger amplitude in variation).

Alternatively, the link between error rates and sequencing coverage could also be an effect of imperfect mapping of reads with sequencing errors if mapping parameters are stringently set. Observed coverage is thus expected to decrease in such regions [10] and may be falsely interpreted as having copy number variation (for genomic sequencing) or reduced expression levels (for transcriptomic sequencing), if these factors are not properly controlled for. However, it should be noted that not all positions with an error prone sequence motif seems to be effected by sequencing coverage bias. For example, the sequence motif “CCNGCC” (and its reverse complement) occurs 22 times in the wolverine mtDNA sequence, and most of these show neither elevated error rates nor decreased sequencing coverage. In order to be able to apply proper controls for SSE bias it is thus important to elucidate why some, but not all, regions with certain sequence motifs show marked increases in sequencing errors. An important development for the future is to develop and utilise more precise methods to control for these biases in bioinformatics pipelines (including base calling, read trimming, error correction and mapping algorithms). Additionally, wet-lab based methods can be improved to produce a more even coverage across regions with different sequence characteristics [41]. As different sequencing technologies show different signatures of bias, combining different types of sequencing data could be a suitable strategy to reduce these effects [20].

## Conclusions

We have reported a striking variation in sequencing coverage across the mitochondrial genome. Reproducibility of coverage patterns within and across species provide evidence that this variation is largely due to intrinsic properties of the DNA sequence, with GC content as an important explanatory factor. Coverage is also related to local levels of sequencing error rates, with peaks in sequencing error also showing marked drops in coverage. Such error peaks are also often associated with certain error prone sequencing motifs. This highlights the importance of controlling for coverage bias when investigating sequencing data for applications such as RNA-Seq, CNV identification or whole genome sequencing.

## Methods

### Samples and sequencing

Sequencing was performed on a HiSeq2000 instrument (Illumina Inc) using TruSeq SBS v3 chemistry, according to the manufacturer’s protocols, with paired-end (PE) reads (length varying from 65 bp to 150 bp) and with insert sizes from 180 bp to 500 bp (Table 1). Base calling was done on the instrument by RTA 1.12.4.2 or 1.13.48 and the resulting .bcl files were converted to Qseq format with OLB-1.9.0 (Illumina Inc) and then demultiplexed, allowing for one mismatch base, and converted to fastq format with CASAVA-1.7.0 (Illumina Inc). Reads that did not pass the quality filtering were excluded.

We used whole genome sequencing data from one mammal (wolverine, *G. gulo*) and one bird (collared flycatcher, *Ficedula albicollis*). Wolverine data were obtained from one female sequenced to a nuclear genome depth of 45X (raw PE data; Ekblom et al., unpublished). DNA from this animal sampled in the province of Jämtland, Sweden in 2010 was extracted from muscle tissue using DNeasy Blood and Tissue kit (Qiagen). In addition, DNA samples were extracted from muscle tissue of 10 unrelated wolverine individuals (all males from different parts of the Scandinavian population) and were sequenced to approximately 10X nuclear coverage each. Flycatcher data were obtained from one focal individual (DNA sampled from muscle tissue with phenol-chloroform extraction) sequenced to 85X as described in [35]. Raw sequence read data is available at NCBI SRA (accession number ERP001377).

### Read mapping

Raw wolverine reads were quality trimmed using ConDeTri [42] and reads from the individual sequenced at high depth were then simultaneously mapped to a published mtDNA reference sequence for this species NCBI Accession number: NC_009685; [32], together with the draft genome assembly (Gugu1.0, R Ekblom unpublished), using BWA [43]. A consensus mtDNA sequence was extracted with samtools and bcftools [44], and manually edited to include also insertions and deletions. The consensus was then used as reference when mapping reads from the additional individuals sequenced at lower coverage. The rationale of mapping reads to both the mitochondrial and the nuclear genome, despite our interest here only lies in mtDNA coverage, relates to the possible occurrence of nuclear copies of mtDNA sequences so-called numts; [45]. Reads originating from such copies will preferentially map to the nuclear genome, when this is included in the reference, and will thus not contribute to the calculation of mtDNA coverage [29].

A collared flycatcher mtDNA reference sequence was first generated by mapping reads from the sequenced individual onto the mitochondrial genome of the closely related species *Ficedula zanthopygia* (NCBI Accession number: NC_015802). From this, a consensus sequence was extracted as above. We found several regions where the sequence differed notably between *F. albicollis* and *F.* z*anthopygia*. Manual editing of the sequence was performed iteratively, with mapping to the new consensus after every update until *albicollis* reads corresponded perfectly to the new consensus sequence. Finally, as for the wolverine, mapping was performed using both the mtDNA consensus sequence and nuclear genome assembly (fAlb15, GI:513788161) together as reference. Pileup files were extracted with samtools and merged between individuals and species. Since mtDNA is a circular molecule and given that BWA only map reads to a linear reference, we introduced a break in the mtDNA molecule. As a consequence, coverage in the end regions became progressively lower towards the start and end nucleotide of the reference. We therefore performed the mapping using a two hundred basepair overlap in the ends of the mtDNA reference sequence. There were also low coverage and high error rates in the region downstream of the cyt-b gene (i.e. the D-loop, or the control region), because of problems of correctly aligning reads to the long repeat region (bp 15,988-16,256). In order to avoid biases introduced by these mapping issues we used only data between position 29 and 15,816 for all downstream analyses of coverage and error rates (trimming away a total of 749 bp).

### Analyses

k-mer counting was done using the jellyfish (ver.1.1.6) software [46], with default settings and k-mer length set to 25 bp. Depth of sequencing (N) was calculated using the formula N = M*(L-k + 1)/L, where M represents the mean k-mer coverage, k is the k-mer length and L is the mean read length [34]. Annotation of the mitochondrial consensus sequences was performed using the Mitos web interface [47] and visualised using CGView [48]. For tests of correlation between GC content and coverage we used coverage from the individual sequenced at high depth and mean GC content in non-overlapping sliding windows of size 50 bp of the mitochondrial genome of the respective species. For the analysis of coverage at individual nucleotides we performed an ANCOVA analysis using nucleotide identity and gene type as categorical variables, and GC content of the surrounding region (50 bp window) as a continuous variable.

We identified regions of the mtDNA sequence that were homologous between wolverine and flycatcher using two-sequence megablast (all hit regions extracted using default settings; http://blast.ncbi.nlm.nih.gov), extracted these using in-house perl scripts and aligned them using MAFFT [49]. Additional data handling and statistical analyses were performed using R 2.15 [50].

### Availability of supporting data

The annotated consensus sequences for the complete mtDNA genomes sequenced in this study are available at NCBI [GenBank: KF415127.1 (wolverine), GenBank: KF293721.1 (collared flycatcher)].

## Electronic supplementary material

Additional file 1: Table S1: Summary output from ANCOVA analysis of depth of coverage of individual bases of the wolverine mtDNA. **Table S2.** Sequencing error rates (percentages), for all possible nucleotide substitutions, in the wolverine mtDNA. **Table S3.** Positions in the wolverine mitochondrial genome with evidence for heteroplasmy in one of the 11 sampled individuals. **Figure S1.** Visualisation of the wolverine mitochondrial DNA sequence. **Figure S2.** k-mer count of wolverine mtDNA sequence read data. **Figure S3.** Comparison of sequencing coverage of the wolverine mtDNA sequence depending on whether read depth or fragment depth were used. We also compare differences in coverage variation for quality trimmed reads versus raw reads. **Figure S4.** Mean depth of sequencing coverage of individual nucleotides. **Figure S5.** Sequencing coverage in three mtDNA regions with similar coverage across all wolverine individuals sequenced. **Figure S6.** Sequencing coverage in three mtDNA regions with different coverage across all wolverine individuals sequenced. **Figure S7.** Relationship between depth of sequencing coverage at orthologous positions of the wolverine and collared flycatcher mtDNA. **Figure S8.** Sequencing coverage for all 11 sampled wolverine individuals. **Figure S9.** Close-up view of error rates around the three positions with a sequencing error rate exceeding 1%. (PDF 1 MB)

## References

[1] Pepke S, Wold B, Mortazavi A (2009). Computation for ChIP-seq and RNA-seq studies. Nat Methods.

[2] Trapnell C, Roberts A, Goff L, Pertea G, Kim D, Kelley DR, Pimentel H, Salzberg SL, Rinn JL, Pachter L (2012). Differential gene and transcript expression analysis of RNA-seq experiments with TopHat and Cufflinks. Nat Protoc.

[3] Yoon S, Xuan Z, Makarov V, Ye K, Sebat J (2009). Sensitive and accurate detection of copy number variants using read depth of coverage. Genome Res.

[4] Yandell M, Ence D (2012). A beginner's guide to eukaryotic genome annotation. Nat Rev Genet.

[5] Schatz MC, Delcher AL, Salzberg SL (2010). Assembly of large genomes using second-generation sequencing. Genome Res.

[6] Treangen TJ, Salzberg SL (2011). Repetitive DNA and next-generation sequencing: computational challenges and solutions. Nat Rev Genet.

[7] Dohm JC, Lottaz C, Borodina T, Himmelbauer H (2008). Substantial biases in ultra-short read data sets from high-throughput DNA sequencing. Nucleic Acids Res.

[8] Aird D, Ross M, Chen W-S, Danielsson M, Fennell T, Russ C, Jaffe D, Nusbaum C, Gnirke A (2011). Analyzing and minimizing PCR amplification bias in Illumina sequencing libraries. Genome Biol.

[9] Sims D, Sudbery I, Ilott NE, Heger A, Ponting CP (2014). Sequencing depth and coverage: key considerations in genomic analyses. Nat Rev Genet.

[10] Nakamura K, Oshima T, Morimoto T, Ikeda S, Yoshikawa H, Shiwa Y, Ishikawa S, Linak MC, Hirai A, Takahashi H, Altaf-Ul-Amin M, Ogasawara N, Kanaya S (2011). Sequence-specific error profile of Illumina sequencers. Nucleic Acids Res.

[11] Benjamini Y, Speed TP (2012). Summarizing and correcting the GC content bias in high-throughput sequencing. Nucleic Acids Res.

[12] van Heesch S, Mokry M, Boskova V, Junker W, Mehon R, Toonen P, de Bruijn E, Shull J, Aitman T, Cuppen E, Guryev V (2013). Systematic biases in DNA copy number originate from isolation procedures. Genome Biol.

[13] Cheung M-S, Down TA, Latorre I, Ahringer J (2011). Systematic bias in high-throughput sequencing data and its correction by BEADS. Nucleic Acids Res.

[14] Davey JW, Cezard T, Fuentes-Utrilla P, Eland C, Gharbi K, Blaxter ML (2012). Special features of RAD Sequencing data: implications for genotyping. Mol Ecol.

[15] Wolf J, Bryk J (2011). General lack of global dosage compensation in ZZ/ZW systems? Broadening the perspective with RNA-seq. BMC Genomics.

[16] Szatkiewicz JP, Wang W, Sullivan PF, Wang W, Sun W (2013). Improving detection of copy-number variation by simultaneous bias correction and read-depth segmentation. Nucleic Acids Res.

[17] Kozarewa I, Ning Z, Quail MA, Sanders MJ, Berriman M, Turner DJ (2009). Amplification-free Illumina sequencing-library preparation facilitates improved mapping and assembly of (G + C)-biased genomes. Nat Methods.

[18] Meacham F, Boffelli D, Dhahbi J, Martin D, Singer M, Pachter L (2011). Identification and correction of systematic error in high-throughput sequence data. BMC Bioinformatics.

[19] Allhoff M, Schonhuth A, Martin M, Costa I, Rahmann S, Marschall T (2013). Discovering motifs that induce sequencing errors. BMC Bioinformatics.

[20] Ross M, Russ C, Costello M, Hollinger A, Lennon N, Hegarty R, Nusbaum C, Jaffe D (2013). Characterizing and measuring bias in sequence data. Genome Biol.

[21] Minoche A, Dohm J, Himmelbauer H (2011). Evaluation of genomic high-throughput sequencing data generated on Illumina HiSeq and Genome Analyzer systems. Genome Biol.

[22] Rasmussen DA, Noor MAF (2009). What can you do with 0.1 x genome coverage? A case study based on a genome survey of the scuttle fly *Megaselia scalaris* (Phoridae). BMC Genomics.

[23] Feldmeyer B, Hoffmeier K, Pfenninger M (2010). The complete mitochondrial genome of Radix balthica (Pulmonata, Basommatophora), obtained by low coverage shot gun next generation sequencing. Mol Phylogenet Evol.

[24] Nabholz B, Jarvis ED, Ellegren H (2010). Obtaining mtDNA genomes from next-generation transcriptome sequencing: a case study on the basal Passerida (Aves: Passeriformes) phylogeny. Mol Phylogenet Evol.

[25] Iorizzo M, Senalik D, Szklarczyk M, Grzebelus D, Spooner D, Simon P (2012). De novo assembly of the carrot mitochondrial genome using next generation sequencing of whole genomic DNA provides first evidence of DNA transfer into an angiosperm plastid genome. BMC Plant Biol.

[26] Miller JM, Malenfant RM, Moore SS, Coltman DW (2012). Short reads, circular genome: skimming SOLiD sequence to construct the bighorn sheep mitochondrial genome. J Hered.

[27] Blower DC, Hereward JP, Ovenden JR (2013). The complete mitochondrial genome of the dusky shark *Carcharhinus obscurus*. Mitochondrial DNA.

[28] Hung C-M, Lin R-C, Chu J-H, Yeh C-F, Yao C-J, Li S-H (2013). The d*e novo* assembly of mitochondrial genomes of the extinct passenger pigeon (*Ectopistes migratorius*) with next generation sequencing. PLoS One.

[29] Samuels DC, Han L, Li J, Quanghu S, Clark TA, Shyr Y, Guo Y (2013). Finding the lost treasures in exome sequencing data. Trends Genet.

[30] Miller FJ, Rosenfeldt FL, Zhang C, Linnane AW, Nagley P (2003). Precise determination of mitochondrial DNA copy number in human skeletal and cardiac muscle by a PCR-based assay: lack of change of copy number with age. Nucleic Acids Res.

[31] Robin ED, Wong R (1988). Mitochondrial DNA molecules and virtual number of mitochondria per cell in mammalian cells. J Cell Physiol.

[32] Arnason U, Gullberg A, Janke A, Kullberg M (2007). Mitogenomic analyses of caniform relationships. Mol Phylogenet Evol.

[33] Walker CW, Vilà C, Landa A, Lindén M, Ellegren H (2001). Genetic variation and population structure in Scandinavian wolverine (*Gulo gulo*) populations. Mol Ecol.

[34] Li R, Fan W, Tian G, Zhu H, He L, Cai J, Huang Q, Cai Q, Li B, Bai Y, Zhang Z, Zhang Y, Wang W, Li J, Wei F, Li H, Jian M, Li J, Zhang Z, Nielsen R, Li D, Gu W, Yang Z, Xuan Z, Ryder OA, Leung FC, Zhou Y, Cao J, Sun X, Fu Y (2010). The sequence and de novo assembly of the giant panda genome. Nature.

[35] Ellegren H, Smeds L, Burri R, Olason PI, Backstrom N, Kawakami T, Kunstner A, Makinen H, Nadachowska-Brzyska K, Qvarnstrom A, Uebbing S, Wolf JBW (2012). The genomic landscape of species divergence in Ficedula flycatchers. Nature.

[36] Sequence assembly with MIRA3, The Definitive Guide[http://mira-assembler.sourceforge.net/docs/DefinitiveGuideToMIRA.html]

[37] Medvedev P, Stanciu M, Brudno M (2009). Computational methods for discovering structural variation with next-generation sequencing. Nat Methods.

[38] Wilhelm BT, Landry J-R (2009). RNA-Seq–quantitative measurement of expression through massively parallel RNA-sequencing. Methods.

[39] Chinnery PF, Thorburn DR, Samuels DC, White SL, Dahl H-HM, Turnbull DM, Lightowlers RN, Howell N (2000). The inheritance of mitochondrial DNA heteroplasmy: random drift, selection or both?. Trends Genet.

[40] Isaacs FJ, Carr PA, Wang HH, Lajoie MJ, Sterling B, Kraal L, Tolonen AC, Gianoulis TA, Goodman DB, Reppas NB, Emig CJ, Bang D, Hwang SJ, Jewett MC, Jacobson JM, Church GM (2011). Precise manipulation of chromosomes in vivo enables genome-wide codon replacement. Science.

[41] Oyola S, Otto T, Gu Y, Maslen G, Manske M, Campino S, Turner D, MacInnis B, Kwiatkowski D, Swerdlow H, Quail M (2012). Optimizing illumina next-generation sequencing library preparation for extremely at-biased genomes. BMC Genomics.

[42] Smeds L, Künstner A (2011). ConDeTri - A content dependent read trimmer for Illumina data. PLoS One.

[43] Li H, Durbin R (2009). Fast and accurate short read alignment with Burrows-Wheeler transform. Bioinformatics.

[44] Li H, Handsaker B, Wysoker A, Fennell T, Ruan J, Homer N, Marth G, Abecasis G, Durbin R, Genome Project Data Processing S (2009). The sequence alignment/map format and SAMtools. Bioinformatics.

[45] Richly E, Leister D (2004). NUMTs in sequenced eukaryotic genomes. Mol Biol Evol.

[46] Marçais G, Kingsford C (2011). A fast, lock-free approach for efficient parallel counting of occurrences of k-mers. Bioinformatics.

[47] Bernt M, Donath A, Jühling F, Externbrink F, Florentz C, Fritzsch G, Pütz J, Middendorf M, Stadler PF (2012). MITOS: improved de novo metazoan mitochondrial genome annotation. Mol Phylogenet Evol.

[48] Grant JR, Stothard P (2008). The CGView server: a comparative genomics tool for circular genomes. Nucleic Acids Res.

[49] Katoh K, Toh H (2008). Recent developments in the MAFFT multiple sequence alignment program. Brief Bioinform.

[50] (2013). R: A Language and Environment for Statistical Computing.

