# Supplementary material for: Patterns of sequencing coverage bias revealed by ultra-deep sequencing of vertebrate mitochondria
Source: BMC Genomics. 2014 Jun 12;15(1):467. doi: 10.1186/1471-2164-15-467 (PMC4070552; doi:10.1186/1471-2164-15-467)
Supplement: Supplementary file 1 — Additional file 1: Table S1: Summary output from ANCOVA analysis of depth of coverage of individual bases of the wolverine mtDNA. Table S2. Sequencing error rates (percentages), for all possible nucleotide substitutions, in the wolverine mtDNA. Table S3. Positions in the wolverine mitochondrial genome with evidence for heteroplasmy in one of the 11 sampled individuals. Figure S1. Visualisation of the wolverine mitochondrial DNA sequence. Figure S2. k-mer count of wolverine mtDNA sequence read data. Figure S3. Comparison of sequencing coverage of the wolverine mtDNA sequence depending on whether read depth or fragment depth were used. We also compare differences in coverage variation for quality trimmed reads versus raw reads. Figure S4. Mean depth of sequencing coverage of individual nucleotides. Figure S5. Sequencing coverage in three mtDNA regions with similar coverage across all wolverine individuals sequenced. Figure S6. Sequencing coverage in three mtDNA regions with different coverage across all wolverine individuals sequenced. Figure S7. Relationship between depth of sequencing coverage at orthologous positions of the wolverine and collared flycatcher mtDNA. Figure S8. Sequencing coverage for all 11 sampled wolverine individuals. Figure S9. Close-up view of error rates around the three positions with a sequencing error rate exceeding 1%. (PDF 1 MB) [file 12864_2014_6152_MOESM1_ESM.pdf]

Additional file 1 – Supplementary Tables and Figures
Table S1-S3, Figure S1-S9

Patterns of Sequencing Coverage Bias Revealed by Ultra-deep Sequencing of Vertebrate Mitochondria

Robert Ekblom*, Linnéa Smeds and Hans Ellegren

Department of Ecology and Genetics, Uppsala University, Uppsala, SE-75236, Sweden

* Corresponding author: Department of Ecology and Genetics, Uppsala University, Norbyvägen 18D, SE-75236 Uppsala, Sweden, Tel: +46 18 471 6468; Email: robert.ekblom@ebc.uu.se

Table S1. Summary output from ANCOVA analysis of depth of coverage of individual bases of the wolverine mtDNA. “GC or AT” is a categorical variable describing if the nucleotide is a G/C or A/T. “Annotation” is a categorical variable describing in what type of sequence the nucleotide located (inter-genic, protein coding gene, rRNA or tRNA). GC content is a continuous variable describing the mean GC content of the 50 bp region surrounding the focal nucleotide.

	df	Sum Sq	Mean Sq	F	p
GC or AT	1	$3.68*10^{10}$	$3.68*10^{10}$	$4.97*10^{3}$	$< 2.2*10^{-16}$
Annotation	3	$3.83*10^{11}$	$1.28*10^{11}$	$1.72*10^{4}$	$< 2.2*10^{-16}$
GC content	1	$4.72*10^{12}$	$4.72*10^{12}$	$6.38*10^{5}$	$< 2.2*10^{-16}$
GC or AT * Annotation	3	$6.74*10^{6}$	$2.25*10^{6}$	0.303	0.823
GC or AT * GC region	1	$9.66*10^{7}$	$9.66*10^{7}$	13.0	0.000305
Annotation * GC region	3	$2.10*10^{7}$	$7.01*10^{6}$	0.947	0.417
GC or AT * Annotation * GC region	3	$7.13*10^{6}$	$2.38*10^{6}$	0.321	0.810
Residuals	15,772	$1.17*10^{11}$	$7.40*10^{6}$		

Table S2. Sequencing error rates (percentages), for all possible nucleotide substitutions, in the wolverine mtDNA

		TO				
		A	T	G	C	total
FROM	A	-	0.035	0.040	0.035	0.115
	T	0.033	-	0.034	0.040	0.108
	G	0.036	0.045	-	0.036	0.117
	C	0.043	0.035	0.036	-	0.114

Table S3. Positions in the wolverine mitochondrial genome with evidence for heteroplasmy in one of the 11 sampled individuals. These six sites all have a high (much higher than 0.1%) proportion of one specific non-reference nucleotide in only one individual ("variation sample"), while all other individuals have a low (less than 0.1%) proportion of non-reference nucleotides at this site.

Position	ref	alt	Individual name of variation sample	Proportion of reads with alternative allele (variation sample)	Proportion of reads with alternative allele (mean of ten other samples)
3554	C	T	JN08-026	2.54%	0.021%
7157	T	C	JS05-293	67.9%	0.015%
7725	G	A	JN08-031	30.6%	0.011%
10428	C	A	JS05-293	17.1%	0.023%
12790	T	C	JN09-020	3.52%	0.015%
14989	G	A	JS10-039	1.29%	0.009%

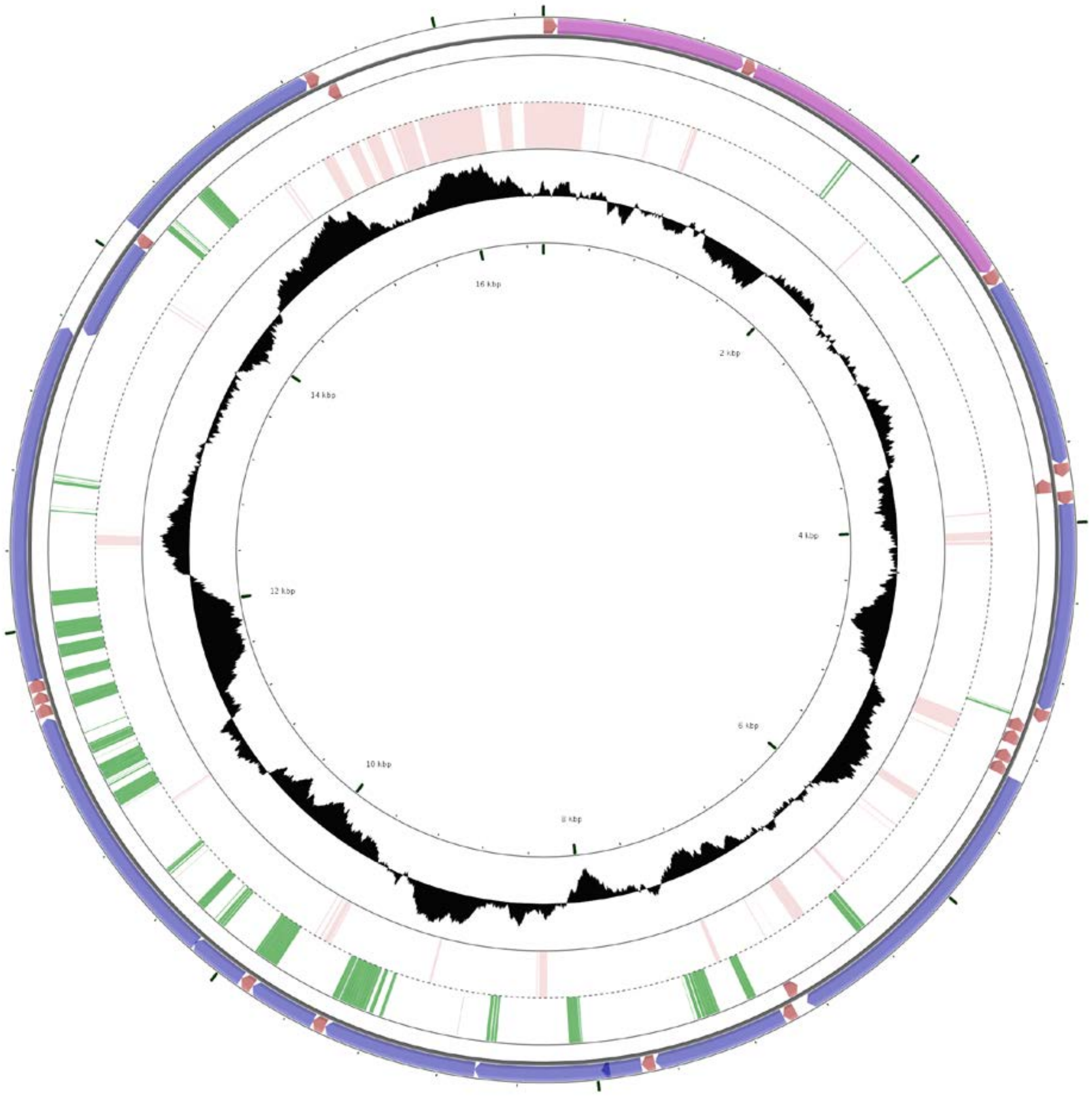

Figure S1. Visualisation of the wolverine mitochondrial DNA sequence. Outer ring: gene annotation (red arrows represent tRNA genes, pink arrows represent rRNA genes and blue arrows represent protein coding genes). Middle ring: high (upper 10th percentile; green) and low (lower 10th percentile; pink) sequence coverage regions. Inner ring: deviation from average GC content (calculated in CGView using a sliding window, with default settings).

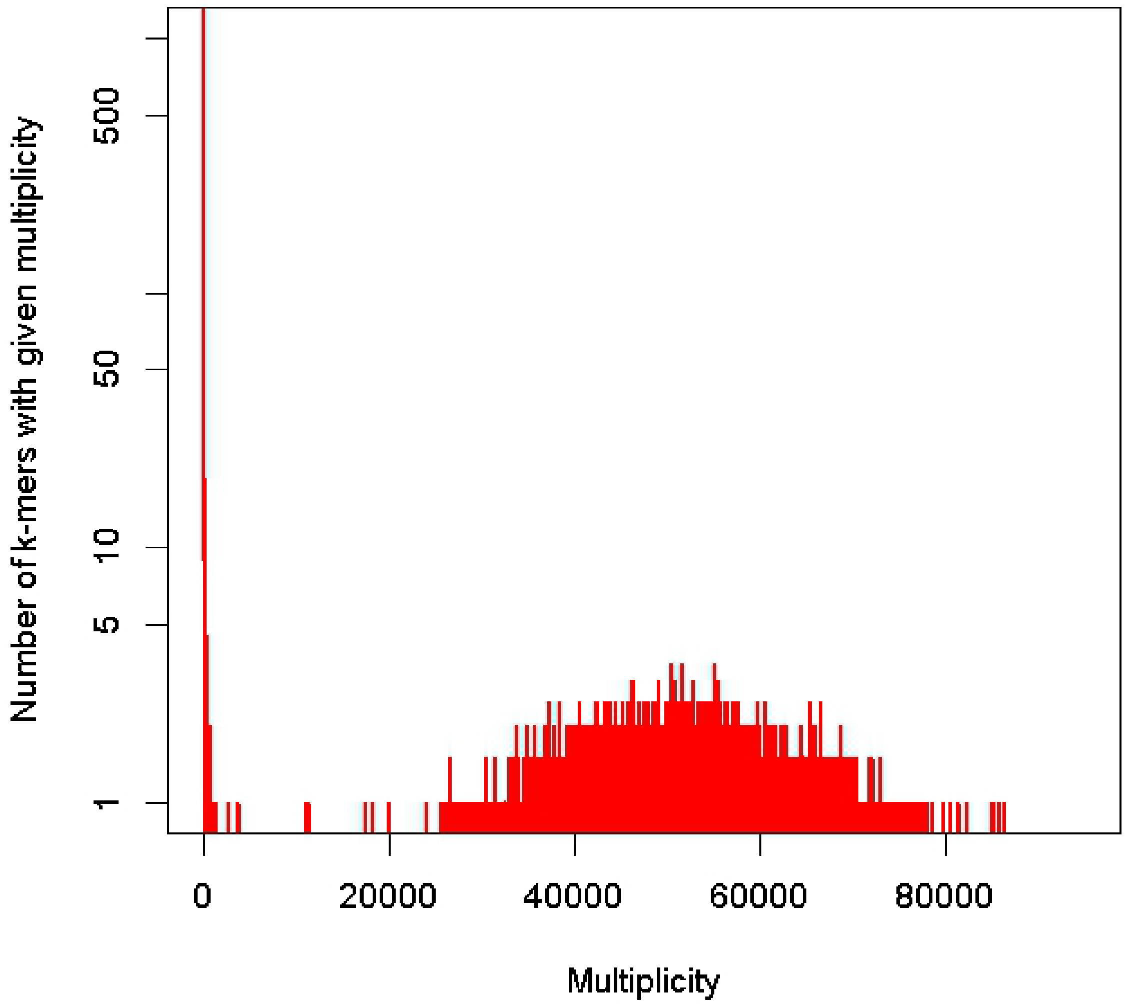

Figure S2. k-mer count of wolverine mtDNA sequence read data (k = 25, y-axis in log scale, truncated).

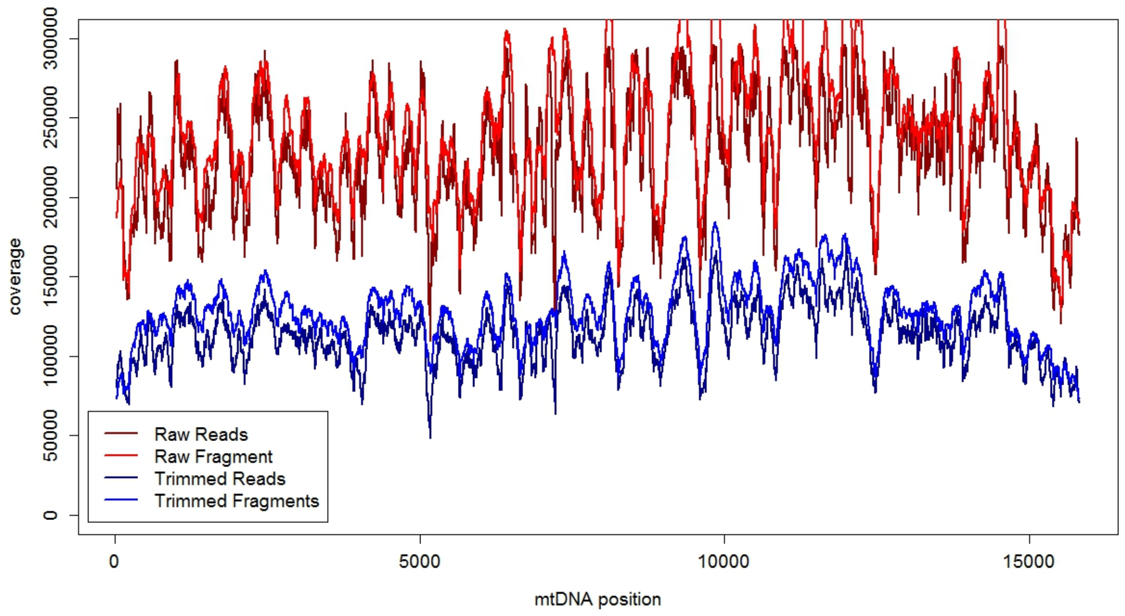

Figure S3. **Comparison of sequencing coverage of the wolverine mtDNA sequence depending on whether read depth or fragment depth were used.** We also compare differences in coverage variation for quality trimmed reads versus raw reads.

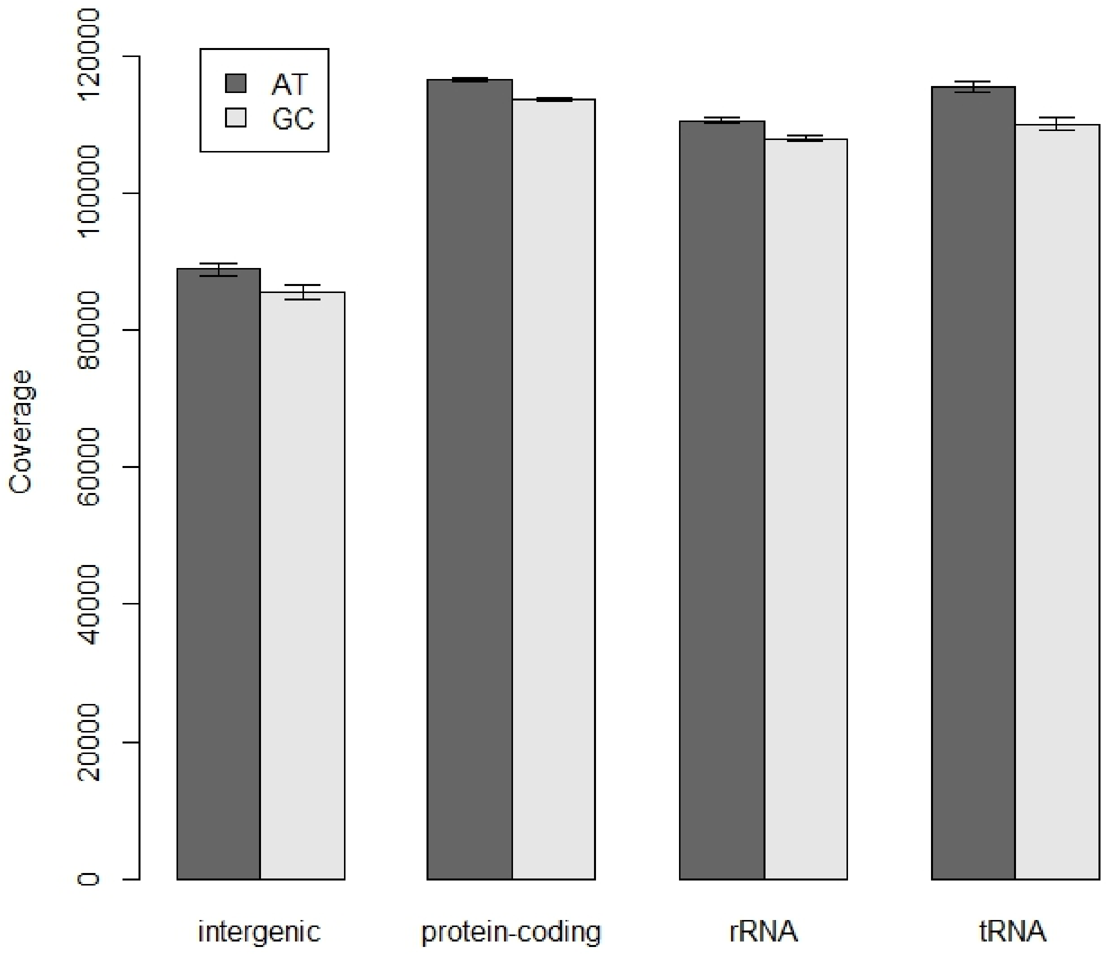

Figure S4. **Mean depth (± SE) of sequencing coverage of individual A/T (dark grey) or G/C (light grey) nucleotides grouped by type of sequence.**

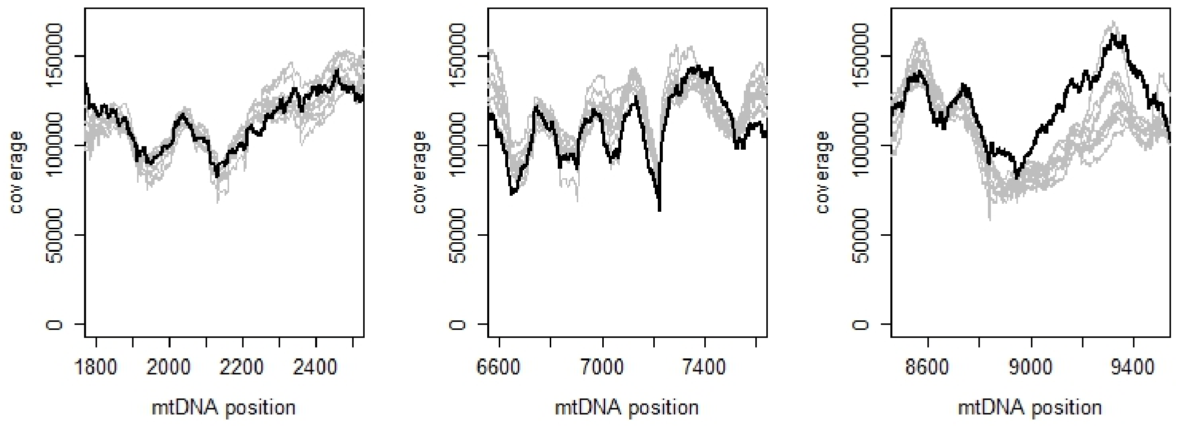

Figure S5. **Sequencing coverage for the main individual (in black) and ten additional sequenced individuals (in grey, scaled to the same mean coverage as the main individual) in three mtDNA regions with similar coverage across all wolverine individuals sequenced.**

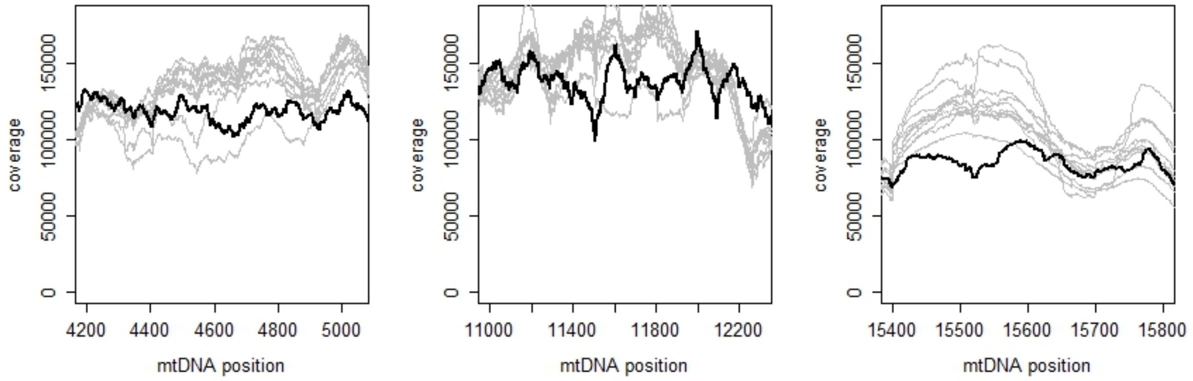

Figure S6. **Sequencing coverage for the main individual (in black) and ten additional sequenced individuals (in grey, scaled to the same mean coverage as the main individual) in three mtDNA regions with different coverage across all wolverine individuals sequenced.**

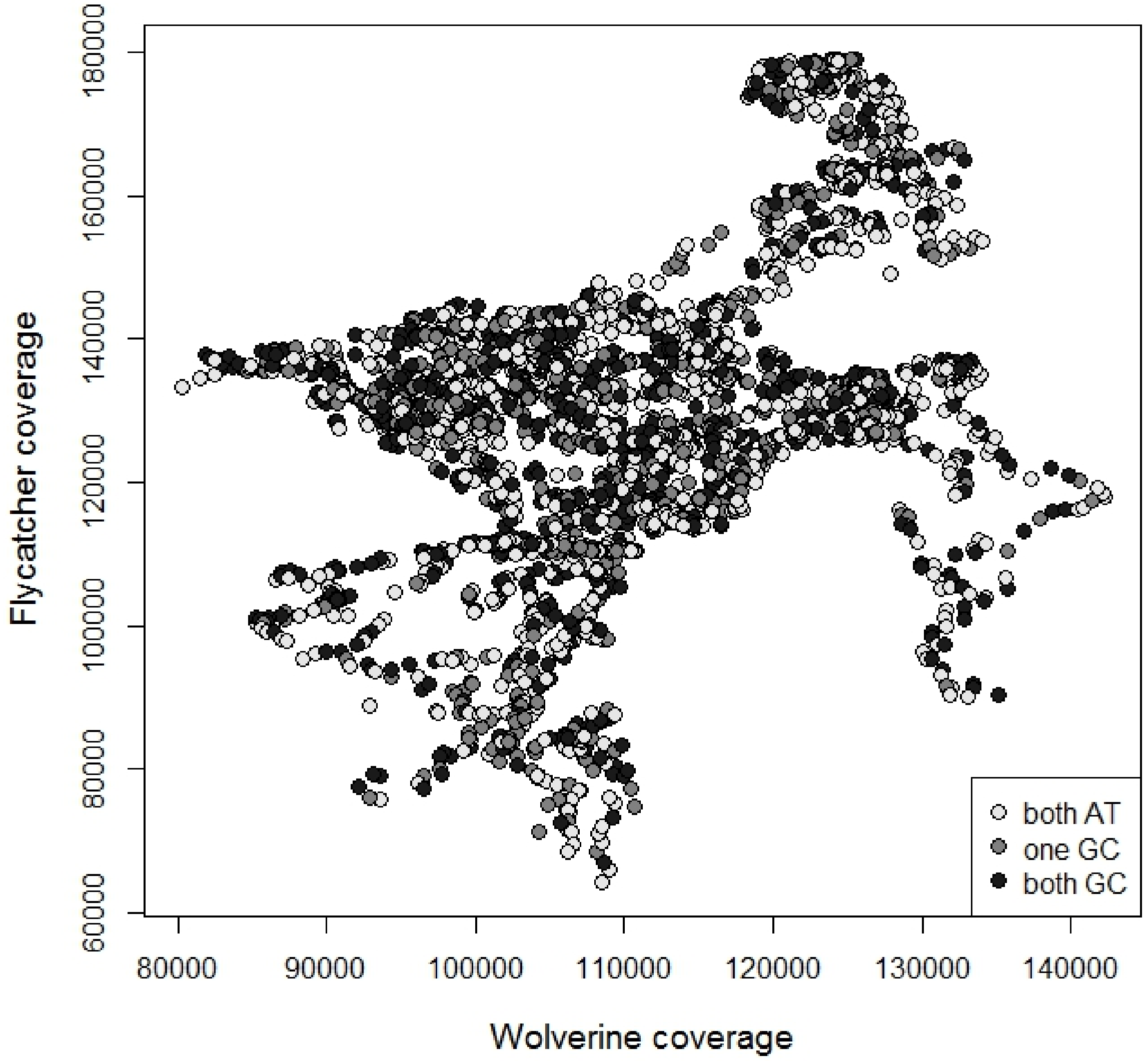

Figure S7. Relationship between depth of sequencing coverage at orthologous positions of the wolverine and collared flycatcher mtDNA. Black circles indicate base pairs where both species have a G or C nucleotide, white indicate that both species have an A or T nucleotide and grey that one species have an A or T and the other a G or C nucleotide.

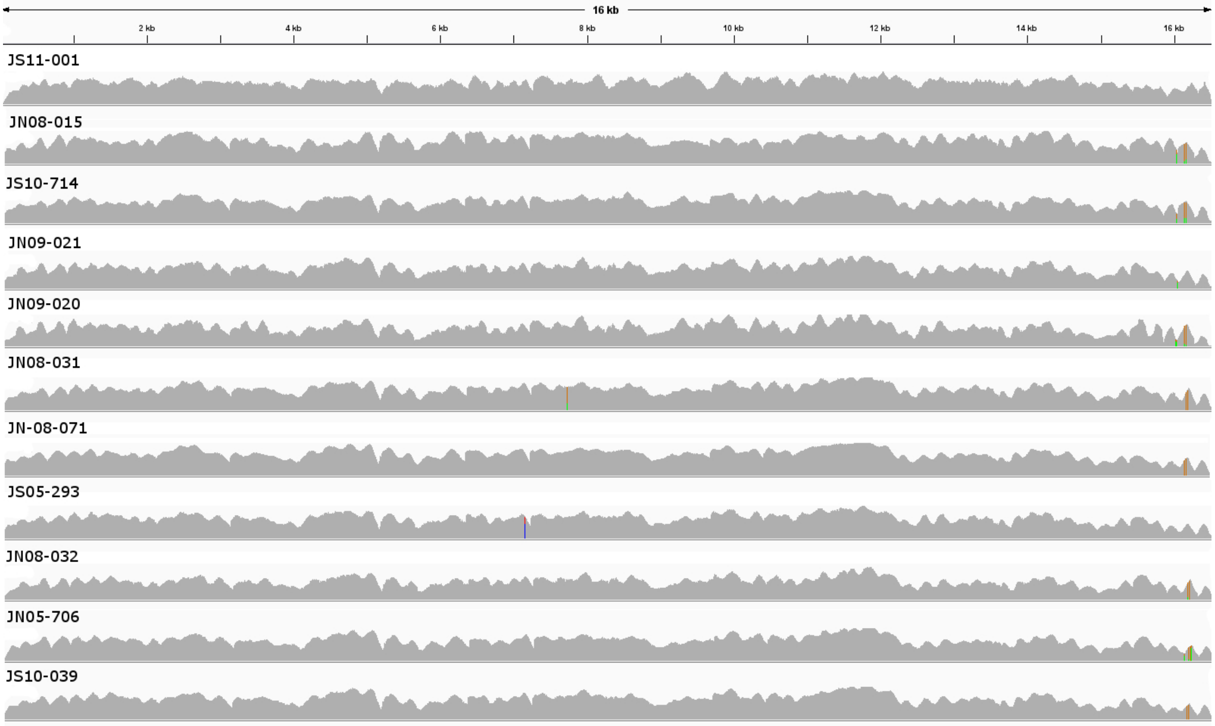

Figure S8. **Sequencing coverage for all 11 sampled wolverine individuals** (sample numbers at the left end of each lane). Two of the heteroplasmic positions (7157 in sample JS05-293 and 7725 in sample JN08-031 are visible as coloured vertical lines. The repeat rich sequence close to the control region (seen as brown and green lines in many individuals close to the rightmost part of the lanes) were excluded in all analyses.

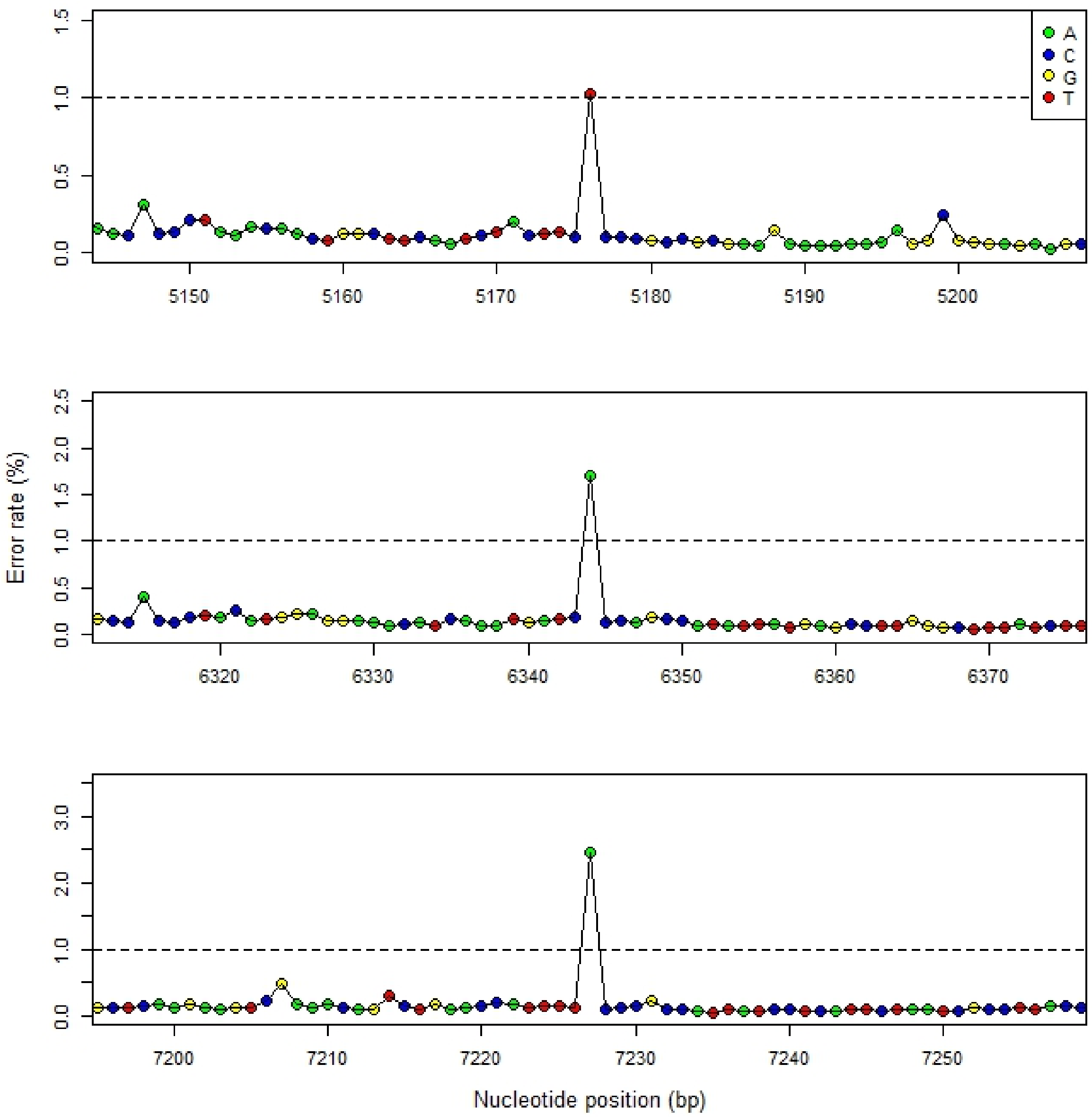

Figure S9. **Close-up view of error rates around the three positions with a sequencing error rate exceeding 1%.** All three positions share the same “CCNGCC” motif directly downstream of the error-prone nucleotide.
